# Development of Enhanced Primer Sets for Detection of Norovirus

**DOI:** 10.1155/2015/103052

**Published:** 2015-01-28

**Authors:** Byoung-Hwa Kong, Sung-Geun Lee, Sang-Ha Han, Ji-Young Jin, Weon-Hwa Jheong, Soon-Young Paik

**Affiliations:** ^1^Department of Biomedical Sciences, College of Medicine, The Catholic University of Korea, 222 Banpo-daero, Seocho-gu, Seoul 137-701, Republic of Korea; ^2^Korea Zoonosis Research Institute, Chonbuk National University, Iksan 570-390, Republic of Korea; ^3^Environmental Infrastructure Research Department, National Institute of Environmental Research, Incheon 404-708, Republic of Korea

## Abstract

Norovirus (NV) is a major viral pathogen that causes nonbacterial acute gastroenteritis and outbreaks of food-borne disease. The genotype of NV most frequently responsible for NV outbreaks is GII.4, which accounts for 60–80% of cases. Moreover, original and new NV variant types have been continuously emerging, and their emergence is related to the recent global increase in NV infection. In this study, we developed advanced primer sets (NKI-F/R/F2, NKII-F/R/R2) for the detection of NV, including the variant types. The new primer sets were compared with conventional primer sets (GI-F1/R1/F2, SRI-1/2/3, GII-F1/R1/F2, and SRII-1/2/3) to evaluate their efficiency when using clinical and environmental samples. Using reverse transcription polymerase chain reaction (RT-PCR) and seminested PCR, NV GI and GII were detected in 91.7% (NKI-F/R/F2), 89.3% (NKII-F/R/R2), 54.2% (GI-F1/R1/F2), 52.5% (GII-F1/R1/F2), 25.0% (SRI-1/2/3), and 32.2% (SRII-1/2/3) of clinical and environmental specimens. Therefore, our primer sets perform better than conventional primer sets in the detection of emerged types of NV and could be used in the future for epidemiological diagnosis of infection with the virus.

## 1. Introduction

Norovirus (NV), belonging to family Caliciviridae, is a major cause of acute viral gastroenteritis [1]. Although symptoms, which typically appear between 12 and 48 h, are generally mild and self-limiting, they can be severe in immunocompromised groups such as infants and the elderly [2, 3]. Viral infection is primarily related to foodborne illness, but person-to-person contact and waterborne outbreaks are also important vehicles for transmission [4–7].

The NV genome is composed of approximately 7.7 kb of single stranded positive sense RNA (+ssRNA), which includes three open reading frames (ORFs): ORF1, ORF2, and ORF3 [8]. Six nonstructural proteins in a polyprotein are encoded by ORF1, including an RNA-dependent RNA polymerase (RdRp) [9]. ORF2 and ORF3 encode major structural capsid protein (VP1) and minor structural capsid protein (VP2), respectively [10]. VP1 consists of a shell domain (S) and two protruding (P) domains [11]. The P1 domain, a protruding flexible hinge region, is located between the S and P2 domains [12]. The P2 domain is a hypervariable region that binds to host cell [13]. The stability of VP1 is increased by VP2, which prevents its degradation [14].

NV is classified into six groups, genogroups I to VI (GI to GVI), based on the amino acid sequences of the RdRp and VP1 [5, 15, 16]. The genogroups GI, GII, and GIV are found in humans [5]. Outbreaks appear more frequently in GI, GII than GIV [17–20]. In particular, GII.4 has emerged continuously every 2-3 years in an evolved form [21]. Consequently, it accounts for 87% of the NV outbreaks that occur globally [22–24].

In the Republic of Korea, NV GII.4 Sydney type emerged between 2012 and 2013, during which time it accounted for 60.4% of NV GII.4 diagnoses [25]. Detection of the NV GII strain is difficult with existing RT-PCR primer sets (GII-F1/R1/F2, SRII-1/2/3) because of the continuous variation of the strain [26, 27]. In addition, GI-F1/R1 primer set does not always have sufficient specificity to detect NV because false-positive detection commonly occurs [28].

Therefore, the aim of this study was to develop primer sets for efficiently detecting NV GI and GII, including detection of newly emerged strains that could not previously be identified with conventional primer sets. Once new primer sets were developed, we evaluated their efficiency using an RT-PCR assay to test clinical and environmental specimens.

## 2. Materials and Methods

### 2.1. Collection of Clinical and Environmental Samples

Two sample types, clinical and environmental specimens, were used for detection of NV GI and GII. Eighty-six unknown samples were used for detection of NV GI. They included 22 clinical samples from Gyeonggi Institute of Health Environment (GIHE) that were originally obtained during 2012 and 2013 and 24 clinical and 40 environmental samples from Waterborne Virus Bank (WAVA) originally obtained from 2006 to 2013. To identify NV GII, we used 134 unknown samples that included 35 clinical samples from GIHE, originally collected during 2012 and 2013, and 32 clinical and 67 environmental samples from WAVA, collected from 2006 to 2013.

All stool specimens were collected from patients who suffered from diarrhea caused by acute gastroenteritis. Environmental specimens were collected from groundwater in the Republic of Korea during June and October 2013. All samples were stored at −80°C until use.

### 2.2. Ethical Clearance

All clinical samples were obtained during the medical treatment of patients with acute gastroenteritis. All patients provided written informed consent, which has been kept on file at the GIHE and WAVA. Human rights were not abused nor were ethical issues encountered during the study. All experimental work and collection of samples were supervised and approved by the Institutional Review Board (IRB) of Songeui Medical Campus, The Catholic University of Korea (approval number MC14SISI0039).

### 2.3. Primer Design

In order to design new primer sets, 37 sequences of NV GI (Table 1) and 52 sequences of NV GII (Table 2) were obtained from NCBI and imported into EditSeq and MegAlign in DNASTAR software (DNASTAR, USA).

We designed 3 candidates for the NV GI primer set and 5 candidates for the NV GII primer set in the conserved regions of ORF 1 and ORF 2. Among them, NKI and NKII (Table 3) which have outstanding efficiency were selected. Inner primers were designed to detect NV from water samples because NV generally presents with a low titer in water [29]. They were constructed based on conserved sites from primer sets NKI-F and NKII-R. We named the inner primers NKI-F2 and NKII-R2 and used them for nested PCR.

### 2.4. Viral RNA Extraction

Viral RNA was extracted from clinical and environmental specimens with the QIAamp viral RNA extraction kit (Qiagen, Germany) according to the manufacturer's instructions. Each viral RNA sample was eluted with 60 *μ*L of elution buffer and stored at −80°C until use in the RT-PCR assay.

### 2.5. RT-PCR and Seminested PCR

Extracted RNA was amplified by both RT-PCR and seminested PCR in S1000 Thermal Cycler (BIO-RAD, Singapore). In RT-PCR, extracted RNA was reverse-transcribed and amplified using the QIAGEN OneStep RT-PCR Kit (Qiagen, Germany) according to the manufacturer's protocol. The total reaction mixture volume of 25 *μ*L contained the following: 5× QIAGEN OneStep RT-PCR buffer (5 *μ*L), 20 pmol primers (1 *μ*L each) (NKI-F/R, NKII-F/R, SRI-1/2, SRII-1/2, GI-F1/R1, and GII-F1/R1), 10 mM dNTP mix (1 *μ*L), enzyme mix (reverse transcriptase and Taq polymerase, 1 *μ*L), extracted viral RNA template (3 *μ*L), and RNase-free water (13 *μ*L) (Welgene, Republic of Korea). RT-PCR conditions were as follows: reverse transcription at 50°C for 30 min, initial PCR activation at 95°C for 15 min, 39 cycles of denaturation at 94°C for 30 s, annealing at each optimal annealing temperature (Tables 4 and 5) for 30 s, extension at 72°C for 1 min, and a final extension at 72°C for 10 min.

For seminested PCR, the RT-PCR product (3 *μ*L), 20 pmol primers (1 *μ*L each) (NKI-F2/R, NKII-F/R2, SRI-2/3, SRII-2/3, GI-F2/R1, and GII-F2/R1), and RNase-free water (15 *μ*L) were added to a Maxime PCR PreMix Kit (*t*-StarTaq) (iNtRON Biotechnology, Republic of Korea). PCR conditions were as follows: initial denaturation at 94°C for 2 min, 20 cycles of denaturation at 94°C for 20 s, annealing at each optimal annealing temperature (Tables 4 and 5) for 10 s, extension at 72°C for 20 s, and a final extension at 72°C for 5 min.

### 2.6. Sequence Analysis of PCR Products

The products of RT-PCR and seminested PCR were analyzed using 2% agarose gel electrophoresis in TAE buffer with DNA SafeStain solution (Lamda Biotech, USA) and were purified in agarose gel with a HiYield Gel/PCR DNA Fragments Extraction Kit (RBC, Taiwan). For sequencing, PCR products were sent to Cosmo Genetech (Republic of Korea), and returned sequences were analyzed using the basic local alignment search tool (BLAST) in NCBI.

### 2.7. Statistical Analysis

An efficiency test of both GI primer sets (GI-F1/R1/F2, SRI-1/2/3, and NKI-F/R/F2) and GII primer sets (GII-F1/R1/F2, SRII-1/2/3, and NKII-F/R/R2) was statistically analyzed using SPSS 20.0 [30].

### 2.8. Nucleotide Sequence Registration

The NV GI and NV GII sequences that were submitted to GenBank in NCBI (http://www.ncbi.nlm.nih.gov/) were isolated from stool and groundwater samples.

## 3. Results

### 3.1. Selection of Primer Sets

Collected NV sequences (37 from NV GI, 52 from NV GII) were aligned using EditSeq and MegAlign in DNASTAR software. Results showed that conserved sequences were selected in ORF1 and ORF2.

From the 3 and 5 respective candidate NV GI and NV GII primer sets that were evaluated for efficiency using clinical samples, the most efficient primer sets were selected and used in this study (Table 3).

### 3.2. Detection of NV GI and GII

**Figure 1:**
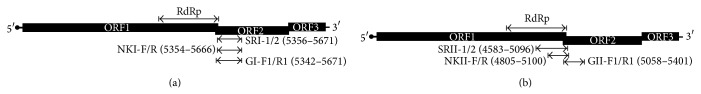
Location of designed primer (NKI F/R, NKII F/R) and conventional primers (SRI-1/2, SRII-1/2, GI-F1/R1, and GII-F1/R1) of NV GI (a) and GII (b).

To evaluate the efficiency of the new primer sets (NKI-F/R/F2, NKII-F/R/R2) designed for this study, we used RT-PCR and compared them to conventional primer sets for NV GI (GI-F1/R1/F2, SRI-1/2/3) and GII (GII-F1/R1/F2, SRII-1/2/3). The ORF1 region was amplified by NKII-F/R/R2 and SRII-1/2/3, while the ORF2 region was amplified by NKI-F/R/F2, SRI-1/2/3, GI-F1/R1/F2, and GII-F1/R1/F2 (Figure 1).

For NV GI, 84 samples were confirmed with GI-F1/R1/F2, SRI-1/2/3, and NKI-F/R/F2 detecting 13, 6, and 22 samples, respectively. For NV GII, 134 samples were identified using GII-F1/R1/F2, SRII-1/2/3, and NKII-F/R/R2 to detect 31, 19, and 53 samples, respectively.

### 3.3. Comparison of Primer Sets

In total, 83 positive samples were identified, including 50 clinical (18 NV GI, 32 NV GII) and 33 environmental (6 NV GI, 27 NV GII) specimens. The most sensitive primer set for NV GI was NKI-F/R/F2, which detected 22/24 positive samples (91.7%). In comparison, the other primers detected 13/24 (54.2%, GI-F1/R1/F2) and 6/24 (25.0%, SRI-1/2/3) samples containing NV GI.

In detecting NV GII, the NKII primer set showed a superior diagnostic yield compared to the other sets. NKIIF/R/R2 identified 53/59 (89.3%) positive NV GII samples, whereas GII-F1/R1/F2 and SRII-1/2/3 identified 31/59 (52.5%) and 19/59 (32.2%), respectively. Sensitivity using each primer set is shown in Table 6 (NV GI) and Table 7 (NV GII). SRI-1/2/3 and SRII-1/2/3 were unsuitable for use with environmental samples because positive samples were not detected.

### 3.4. Sequence Analysis

Results of BLAST sequence analysis indicated that some of the PCR products were amplified using several primers (GI-F1/R1/F2, GII-F1/R1/F2, and SRI-1/2/3) which were shown as false positives of NV, whereas NKI-F/R/F2, NKII-F/R/R2, and SRII-1/2/3 did not detect false positives for NV. However, NKI-F/R/F2 and SRI-1/2/3 did detect NV GII in some samples. Where NV GII was detected, it had similar sequences, which were as follows: AB290150 (NKI-F/R/F2), FJ383875 (NKI-F/R/F2, SRI-1/2/3), KF289337 (SRI-1/2/3), and KF509946 (NKI-F/R/F2, SRI-1/2/3).

Genotypes of NV GI.3, GI.4, GI.6, GI.8, and GI.15 and NV GII.2, GII.3, GII.4, GII.6, GII.13, GII.16, GII.17, and GII.21 were identified using GI and GII primer sets, respectively (Tables 6 and 7). Using all primer sets, 17 samples containing the NV GII.4 variant were identified. NKII-F/R/R2, SRII-1/2/3, and GII-F1M/R1M/F3M detected NV GII.4 variant type in 16, 12, and 10 samples, respectively.

From positive samples, amplified sequences obtained using NKIF/R/F2 and NKII-F/R/R2 were registered in NCBI. The deposited accession numbers were as follows: KJ742428, KJ742429, KJ742430, KJ742431, KJ742432, KJ742433, KJ742434, KJ742435, KJ742436, KJ742437, KJ742438, KJ742439, KJ742440, KM017944, KM017945, KM017946, KM017947, KM017948, KM017949, KM017950, KM017951, KM017952, KM017953, KM017954, KM017955, KM017956, KM017957, KM017958, and KM017959.

## 4. Discussion

In the USA, estimated 570–800 people die annually from outcomes associated with NV. Outbreaks of the virus occur regularly, and new variant strains are identified worldwide every 2 to 3 years [3, 21, 31, 32]. Methods for detection of NV include RT-PCR, the enzyme-linked immunosorbent assay (ELISA), and transmission electron microscopy (TEM) [33, 34]. In previous studies, virus concentrations up to 10^2^ virus-copies/mL were detected using RT-PCR compared to 10^5^ virus-copies/mL with other methods [33]. The widespread use of RT-PCR for NV detection can be attributed to its superior sensitivity compared to other methods [35, 36]. For example, it is the recommended method for detection of NV in contaminated water, in which NV commonly exists at 4.91× 10^2^–3.51 ×10^3^ copies/mL [29]. However, in order to minimize outbreaks and prevent potential deaths, improved primer sets are required to accurately detect original and variant strains of NV.

It is possible that the primer sets used for detection of NV are different in each laboratory because a standard method for detection has not yet been established [37]. In previously published work, the primer sets GI-F1/R1/F2 and GII-F1/R1/F2, SRI-1/2/3 and SRII-1/2/3 have typically been used to detect NV [38, 39]. Indeed, GI-F1/R1/F2 and GII-F1/R1/F2 are recommended for detection of NV by the Centers for Disease Control and Prevention's (CDC) of Republic of Korea (http://www.cdc.go.kr/CDC/contents/CdcKrContentView.jsp?cid=18302&menuIds=HOME001-MNU1175MNU834-MNU0839). Lee et al. (2011) used SRI-1/2/3, SRII-1/2/3, GI-F1/R1/F2, and GII-F1/R1/F2 primer sets when testing water samples collected in 2008 near groundwater in Republic of Korea [40]. They found that 117 sites were contaminated with NV GI and GII, and the study indicated that NV could more efficiently be detected with GI-F1/R1/F2 (35 samples) and GII-F1/R1/F2 (55 samples) compared to SRI-1/2/3 (27 samples) and SRII-1/2/3 (41 samples) [40]. Similarly, in the present study, the performance of SRI-1/2/3 and SRII-1/2/3 in detection of NV was inferior to other primer sets. In particular, SRI-1/2/3 and SRII-1/2/3 entirely failed to detect NV in water. Conversely, the majority of positive NV samples were detected using NKI-F/R/F2 (91.7%) and NKII-F/R/R2 (89.3%). We postulate that because the sequences of NKI-F/R/F2 and NKII-F/R/R2 were collected from 1968 to 2013, their design contained more conserved sequences in comparison to the GI-F1/R1/F2, GII-F1/R1/F2, SRI, and SRII primer sets and this could explain their superior performance.

NV GII.4 is known to be the most prevalent strain of NV [41], and since 2012 a new variant of NV GII.4 has caused many cases of gastroenteritis [42]. In detecting NV GII.4, an RT-PCR assay using a GII-F1/R1/F2 primer set or another forward primer (Cog2F, F2 FB, GV21) with a GII-F1/R1/F2 reverse primer has typically been used [25, 42–44]. However, these primer sets were designed before 2005 and are not suitable for detection of new NV mutant types that have continuously emerged since, such as the 2012 Sydney strain. Their unsuitability is probably explained by differences in sequence between the primers and the new variant types [45, 46].

In this study, NKII-F/R/R2, GII-F1/R1/F2, and SRII were used to detect the NV GII.4 variant. In total, 17 NV GII variant samples were confirmed using NKII-F/R/R2, SRII-1/2/3, and GII-F1/R1/F2. They detected 16, 12, and 10 NV GII samples, respectively. The GII-F1/R1/F2 primer set has been widely used for detection of NV GII.4 [42–44, 47]. However, in previous research, the efficiency of this primer set for detecting NV GII.4 was relatively low [25, 41]. Therefore, GII-F1/R1/F2 may be an inappropriate method for detection of the NV GII.4 variant.

## 5. Conclusions

In our study, we showed that the NKI-F/R/F2 and NKII-F/R/R2 primer sets could be important for epidemiological diagnosis of NV in the laboratory because of their superior performance compared to other primer sets. Where contamination with NV was suspected, these primer sets could be applied to specimens taken from water sample with a low titer or clinical samples with a high titer. Additionally, our newly developed primer sets can be used to detect variant types of NV. Therefore, we recommend the use of NKI-F/R/F2 and NKII-F/R/R2 in future research.

## Figures and Tables

**Table 1:** Description of NV GI sequences for genetic analysis.

Accession number (GenBank)	Genotype	Strain	Accession number (GenBank)	Genotype	Strain
EU085529	GI.1	P774.Delsjo2004/Gothenburg/Sweden	KF039731	GI.1	CH4XO533/2009/USA
FJ515294	GI.2	Leuven/2003/BEL	KF039732	GI.1	CHA5A010/2009/USA
JN176918	GI.2	Roosendaal029/2006/NL	KF039733	GI.1	CHA9A004_20110426/2011/USA
JN183159	GI.9	S48/2008/Lilla	KF039734	GI.1	CHA6A014/2009/USA
JN183161	GI.7	S24/2008/Lilla Edet	KF039735	GI.1	CHA3A007/2008/USA
JN603244	GI.3	S29/2008/Lilla Edet/Sweden	KF039736	GI.1	CHA7A011/2010/USA
JN603245	GI.4	S50/2008/Lilla Edet/Sweden	KF039737	GI.1	CHA6A003_20091104/2009/USA
JQ388274	GI.6	Kingston/ACT160D/2010/AU	KF306212	GI.2	Jingzhou/2013401/CHN
JQ743331	GI.4	2000	KF429761	GI.1	8MoIIIL/1972/USA
JQ743332	GI.2	1999	KF429765	GI.1	8W/1968/USA
JQ911594	GI	10360/2010/VNM	KF429770	GI.1	8McIII/1973/USA
JX023285	GI.1	8FIIa/1968/USA	KF429773	GI.1	8CKIIIc/1974/USA
KC998959	GI.6	TCH-099/USA/2003	KF429774	GI.1	8UIIIf/1973/USA
KF039725	GI.1	CHA7A009/2010/USA	KF429783	GI.1	8K/1979/USA
KF039726	GI.1	/CHA6A003_20091031/2009/USA	KF429789	GI.1	8MC/1978/USA
KF039727	GI.1	CHA2A014/2008/USA	KF586507	GI.9	CAIQ12110628
KF039728	GI.1	CHA2A014/2008/USA	L07418		
KF039729	GI.1	CHA6A007/2010/USA	M87661		
KF039730	GI.1	CHA9A004_20110419/2011/USA			

**Table 2:** Description of NV GII sequences for genetic analysis.

Accession number (GenBank)	Genotype	Strain	Accession number (GenBank)	Genotype	Strain
AB447433	GII.4	Aormori/2006/JP	JQ320072	GII.2	NF2002/USA/2002
AB541319	GII.4	Osaka/2007/JP	JX445152	GII.4	AlbertaE131/2004/CA
AB541321	GII.4	Osaka2/2007/JP	JX445153	GII.4	AlbertaEI142/2006/CA
AB541362	GII.4	Toyama5/2008/JP	JX445157	GII.4	AlbertaEI513/2006/CA
AB543808	GII.4	FUMI/2010/JP	JX445159	GII.4	AlbertaEI009/2008/CA
AB662873	GII-2	OC09104/2009/JP	JX445161	GII.4	AlbertaEI210/2008/CA
AY485642	GII.4	Langen 1061/2002/GER	JX445168	GII.4	AlbertaEI388/2008/CA
AY502023	GII.4	Farmington Hills/2002/USA	JX459900	GII.4	NSW882J/2011/AU
DQ658413	GII.4	MD-2004/2004USA	JN899245	GII.21	Salisbury150/2011/USA
DQ078814	GII.4	Hunter504D/2004/AU	JX459907	GII.4	NSW3309/2012/AU
EF202588	GII.4	Toronto SK/2005/CAN	JX459908	GII.4	NSW0514/2012/AU
EU310927	GII.4	TCH186 2002 US	JX846924	GII.3	HK71/1978/CHN
EF684915	GII.4	Shellharbour NSW696T/2006/AUS	JX846925	GII.2	KL109/1978/MYS
FJ537135	GII.4	CHDC2094/1974/US	JX846926	GII.7	CHDC3936/1988/USA
GQ645366	GII.4	NSW3639/2008/AUS	JX989075	GII.6	GZ2010-L96/Guangzhou/CHN 2011
GQ845368	GII.4	NSW505G 2007 AUS	KC175323	GII.4	Hong kong CUHK3630/2012/CHN
GQ845369	GII.4	Armidale NSW3901/2008/AU	KC464499	GII.12	CGMH41/2010/TW
GU017907	GII.14	8594/Maizuru/2008/ JPN	KC464505	GII.2	CGMH47/2011/TW
GU134965	GII.7	1738/2009/USA	KC576910	GII.6	S9c/1976/SEN
GU445325	GII.4	New Orleans1805/2009/USA	KC597138	GII.2	CHDC2596/1975/USA
GU969058	GII.13	8679/Maizuru/2008 JPN	KC597139	GII.17	C142/1978/GUF
GU980585	GII.3	CBNU1/2006/KOR	KC597140	GII.3	NIHIC8.1/2011/USA
HQ664990	GII.12	HS206/2010/USA	KF306213	GII.3	Jingzhou/2013402/CHN
JN400623	GII.4	CGMH25/2010/TW	KF429764	GII.1	21K1/1972/USA
JN595867	GII.4	Ascension208/2010	KF429769	GII.2	SnowMountRS/1975/USA
JN797508	GII.1	HawaiiTD/1974/USA	X86557	GII.4	Lordsdale virus

**Table:** (a) GI

NKI primer set																						
Primer	NKI-F (forward)																					
	G	T	A	A	A	T	G	A	T	G	A	T	G	G	C	G	T	C	T	A	A	

M87661 (GI)	.	.	.	.	.	.	.	.	.	.	.	.	.	.	.	.	.	.	.	.	.	0
KF039732 (GI.1)	.	.	.	.	.	.	.	.	.	.	.	.	.	.	.	.	.	.	.	.	.	0
KF306212 (GI.2)	.	.	.	.	.	.	.	.	.	.	.	.	.	.	.	.	.	.	.	.	.	0
JN603244 (GI.3)	.	.	.	.	.	.	.	.	.	.	.	.	.	.	.	.	.	.	.	.	.	0
JN603245 (GI.4)	.	.	.	.	.	.	.	.	.	.	.	.	.	.	.	.	.	.	.	.	.	0
JQ388274 (GI.6)	.	.	.	.	.	.	.	.	.	.	.	.	.	.	.	.	.	.	.	.	.	0
JN183161 (GI.7)	.	.	.	.	.	.	.	.	.	.	.	.	.	.	.	.	.	.	.	.	.	0
KF586507 (GI.9)	.	.	.	.	.	.	.	.	.	.	.	.	.	.	.	.	.	.	.	.	.	0

Primer	NKI-R (reverse)																					
	A	C	C	C	A	D	C	C	A	T	T	R	T	A	C	A	T	Y	T	G		

M87661 (GI)	.	.	.	.	.	.	.	.	.	.	.	.	.	.	.	.	.	.	.	.	0	
KF039732 (GI.1)	.	.	.	.	.	.	.	.	.	.	.	.	.	.	.	.	.	.	.	.	0	
KF306212 (GI.2)	.	.	.	.	.	.	.	.	.	.	.	.	.	.	.	.	.	.	.	.	0	
JN603244 (GI.3)	.	.	.	.	.	G	.	.	.	.	.	.	.	.	.	.	.	.	.	.	1	
JN603245 (GI.4)	.	.	.	.	.	.	.	.	.	.	.	.	.	.	.	.	.	.	.	.	0	
JQ388274 (GI.6)	.	.	.	.	.	.	.	.	.	.	.	.	.	.	.	.	.	.	.	.	0	
JN183161 (GI.7)	.	.	.	.	.	.	.	.	.	.	.	.	.	.	.	.	.	.	.	.	0	
KF586507 (GI.9)	.	.	.	.	.	.	.	.	.	.	.	.	.	.	.	.	.	.	.	.	0	

**Table:** (b) GII

NKII primer set																						
Primer	NKII-F (forward)																					
	C	T	Y	A	G	G	C	A	R	A	T	G	T	A	C	T	G	G	A	C	Y	

X86557 (GII)	.	.	.	.	.	.	.	.	.	.	.	.	.	.	.	.	.	.	.	.	.	0
JN797508 (GII.1)	.	.	.	.	.	A	.	.	.	.	.	.	.	.	T	.	.	.	.	.	.	2
KF429769 (GII.2)	.	.	.	.	.	.	.	.	.	.	.	.	.	.	.	.	.	.	.	.	.	0
KF306213 (GII.3)	.	.	.	.	.	A	.	.	.	.	.	.	.	.	T	.	.	.	.	.	.	2
JX459907 (GII.4)	.	.	.	.	.	.	.	.	.	.	.	.	.	.	.	.	.	.	.	.	.	0
KC464499 (GII.12)	.	.	.	.	.	A	.	.	.	.	.	.	.	.	T	.	.	.	.	.	.	2
KC597139 (GII.17)	.	.	.	.	.	.	.	.	.	.	.	.	.	.	.	.	.	.	.	.	.	0
JN899245 (GII.21)	.	.	.	.	.	A	.	.	.	.	.	.	.	.	.	.	.	.	.	.	.	1

Primer	NKII-R (reverse)																					
	T	C	G	A	C	G	C	C	A	T	C	T	T	C	A	T	T	C	A	C		

X86557 (GII)	.	.	.	.	.	.	.	.	.	.	.	.	.	.	.	.	.	.	.	.	0	
JN797508 (GII.1)	.	.	.	.	.	.	.	.	.	.	.	.	.	.	.	.	.	.	.	.	0	
KF429769 (GII.2)	.	.	.	.	.	.	.	.	.	.	.	.	.	.	.	.	.	.	.	.	0	
KF306213 (GII.3)	.	.	.	.	.	.	.	.	.	.	.	.	.	.	.	.	.	.	.	.	0	
JX459907 (GII.4)	.	.	.	.	.	.	.	.	.	.	.	.	.	.	.	.	.	.	.	.	0	
KC464499 (GII.12)	.	.	.	.	.	.	.	.	.	.	.	.	.	.	.	.	.	.	.	.	0	
KC597139 (GII.17)	.	.	.	.	.	.	.	.	.	.	.	.	.	.	.	.	.	.	.	.	0	
JN899245 (GII.21)	.	.	.	.	.	.	.	.	.	.	.	.	.	.	.	.	.	.	.	.	0	

**Table 4:** Information of NV GI primer sets.

Primers	Sequence (5′-3′)^a^	Location^b^	Region	Annealing temperature (°C)	Polarity	Reference
NKI-F	GTA AAT GAT GAT GGC GTC TAA	5354–5373	Capsid	51	+	This study
NKI-R	ACC CAD CCA TTR TAC ATY TG	5649–5668			−	
NKI-F2	GAT GGC GTC TAA GGA CGC	5363–5380			+	

GI-F1	CTG CCC GAA TTY GTA AAT GAT GAT	5342–5365	Capsid	55	+	[26]
GI-R1	CCA ACC CAR CCA TTR TAC ATY TG	5649–5671			−	
GI-F2	ATG ATG ATG GCG TCT AAG GAC GC	5358–5380			+	

SRI-1	CCA ACC CAR CCA TTR TAC AT	5652–5671	Capsid	50	−	[27]
SRI-2	AAA TGA TGA TGG CGT CTA	5356–5373			+	
SRI-3	AAA AYR TCA CCG GGK GTA T	5578–5596			−	

^a^The means of alphabet sequence are the following: Y = C, T; R = A, G; K = G, T; D = A, G, T.

^b^Location is based on accession number M87661 (Norwalk virus).

**Table 5:** Information of NV GII primer sets.

Primers	Sequence (5′-3′)^a^	Location^b^	Region	Annealing temperature (°C)	Polarity	Reference
NKII-F	CTY AGG CAR ATG TAC TGG ACY	4805–4825	RdRp	55	+	This study
NKII-R	TCG ACG CCA TCT TCA TTC AC	5081–5100			−	
NKII-R2	GGA GCC AGA TTG CGA TCG C	5060–5078			−	

GII-F1	GGG AGG GCG ATC GCA ATC T	5049–5067	Capsid	55	+	[26]
GII-R1	CCR CCI GCA TRI CCR TTR TAC AT	5367–5389			−	
GII-F2	TTG TGA ATG AAG ATG GCG TCG ART	5079–5102			+	

SRII-1	CGC CAT CTT CAT TCA CAA A	5078–5096	RdRp	50	−	[27]
SRII-2	TWC TCY TTY TAT GGT GAT GAT GA	4583–4605			+	
SRII-3	TTW CCA AAC CAA CCW GCT G	4767–4785			−	

^a^The means of alphabet sequence are the following: Y = C, T; R = A, G; I = inosine; W = A, T.

^b^Location is based on accession number X86557 (Lordsdale virus).

**Table 6:** Sensitivity of NV GI using each primer set.

Positive specimens	NKI F/R/F2	GI F1/R1/F2	SRI 1/2/3
GI.3	3	3	—
GI.4	6	2	4
GI.6	5	4	1
GI.8	1	—	1
GI.15	1	—	—
GI	1	1	—
Clinical samples (*n* = 18)	17 (94.4%)^*^	10 (58.8%)	6 (33.3%)

GI.3	4	3	—
GI.6	1	—	—
Environmental samples (*n* = 6)	5 (83.3%)	3 (50.0%)	0 (0%)

Total (*n* = 24)	22 (91.7%)	13 (54.2%)	6 (25.0%)

All *P* values < 0.05 are by *t*-test except for environmental samples (GI F1/R1/F2 and NKI F/R/F2).

Environmental samples were not detected by SRI 1/2/3 primer.

^*^Percentage is NV detection/NV positive ratio.

**Table 7:** Sensitivity of NV GII using each primer set.

Positive specimens	NKII F/R/R2	GII F1/R1/F2	SRII 1/2/3
GII.2	2	1	1
GII.3	1	—	1
GII.4	18	11	12
GII.6	2	1	
GII.13	—	1	—
GII.16	1	1	—
GII.17	4	2	4
GII.21	1	—	—
GII	1	1	1
Clinical samples (*n* = 32)	30 (93.8%)^*^	18 (56.3%)	19 (59.4%)

GII.4	22	11	—
GII	1	2	—
Environmental samples (*n* = 27)	23 (85.7%)	13 (48.1%)	0 (0%)

Total (*n* = 59)	53 (89.3%)	31 (52.5%)	19 (32.2%)

All *P* values < 0.05 are by *t*-test.

Environmental samples were not detected by SRII 1/2/3 primer set.

^*^Percentage is NV detection/NV positive ratio.
